# Central and Peripheral Effects of Urotensin II and Urotensin II-Related Peptides on Cardiac Baroreflex Sensitivity in Trout

**DOI:** 10.3389/fnins.2017.00051

**Published:** 2017-02-10

**Authors:** Frédéric Lancien, Gilmer Vanegas, Jérôme Leprince, Hubert Vaudry, Jean-Claude Le Mével

**Affiliations:** ^1^Institut National de la Santé et de la Recherche Médicale UMR1101, Laboratoire de Neurophysiologie, SFR ScInBioS, Université de Brest, Faculté de Médecine et des Sciences de la SantéBrest, France; ^2^Institut National de la Santé et de la Recherche Médicale U982, UA Centre National de la Recherche Scientifique, Différenciation et Communication Neuronale et Neuroendocrine, Normandie UniversitéRouen, France

**Keywords:** urotensin II, urotensin II-related peptide 1, urotensin II-related peptide 2, intracerebroventricular injection, peripheral injection, baroreflex, autonomic nervous system, trout

## Abstract

The baroreflex response is an essential component of the cardiovascular regulation that buffers abrupt changes in blood pressure to maintain homeostasis. Urotensin II (UII) and its receptor UT are present in the brain and in peripheral cardiovascular tissues of fish and mammals. Intracerebroventricular (ICV) injection of UII in these vertebrates provokes hypertension and tachycardia, suggesting that the cardio-inhibitory baroreflex response is impaired. Since nothing is known about the effect of UII on the cardiac baroreflex sensitivity (BRS), we decided to clarify the changes in spontaneous BRS using a cross spectral analysis technique of systolic blood pressure (SBP) and R–R interval variabilities after ICV and intra-arterial (IA) injections of trout UII in the unanesthetized trout. We contrasted the effects of UII with those observed for the UII-related peptides (URP), URP1 and URP2. Compared with vehicle-injected trout, ICV injection of UII (5–500 pmol) produced a gradual increase in SBP, a decrease in the R–R interval (reflecting a tachycardia) associated with a dose-dependent reduction of the BRS. The threshold dose for a significant effect on these parameters was 50 pmol (BRS; −55%; 1450 ± 165 ms/kPa vs. 3240 ± 300 ms/kPa; *P* < 0.05). Only the 500-pmol dose of URP2 caused a significant increase in SBP without changing significantly the R–R interval but reduced the BRS. IA injection of UII (5–500 pmol) caused a dose-dependent elevation of SBP. Contrasting with the ICV effects of UII, the R–R interval increased (reflecting a bradycardia) up to the 50-pmol dose while the BRS remained unchanged (50 pmol; 2530 ± 270 ms/kPa vs. 2600 ± 180 ms/kPa; *P* < 0.05). Nonetheless, the highest dose of UII reduced the BRS as did the highest dose of URP1. In conclusion, the contrasting effect of low picomolar doses of UII after central and peripheral injection on the BRS suggests that only the central urotensinergic system is involved in the attenuation of the BRS. The limited and quite divergent effects of URP1 and URP2 on the BRS, indicate that the action of UII is specific for this peptide. Further studies are required to elucidate the site(s) and mechanisms of action of UII on the baroreflex pathways. Whether such effects of central UII on the BRS exist in mammals including humans warrants further investigations.

## Introduction

Urotensin II (UII) is a cyclic neuropeptide that was originally isolated and purified from the caudal neurosecretory system of the teleost fish *Gillichthys mirabilis* and later characterized in mammals (Pearson et al., 1980; Vaudry et al., 2010, 2015). UII belongs to a family of structurally related peptides that includes UII and UII-related peptides (URPs) called URP, URP1, and URP2. In the teleost lineage, the four peptides are present but only two of them, UII and URP, are found in tetrapods (Tostivint et al., 2013). UII and URP bind to an ancestral (UII) receptor, termed UT, and the two peptides activate this receptor with similar potency. In teleost fish, which possess different UT subtypes (Tostivint et al., 2014), UII, URPs, and UT are present in the central nervous system (CNS) i.e., in the brainstem and spinal cord (Lu et al., 2006; Nobata et al., 2011; Parmentier et al., 2011; Quan et al., 2015) and UT has been identified in various peripheral organs including the cardiovascular system (Lu et al., 2006). In mammals, UII and URP genes are mostly and differentially expressed in cholinergic motor neurons of the brainstem and spinal cord, and UII and UT immunoreactivity are present in neurons of the brainstem involved in cardiovascular functions (Dun et al., 2001; Pelletier et al., 2002; Jégou et al., 2006). UII, URP, and UT mRNAs are also differentially expressed in peripheral tissues, including notably the cardiovascular system (Ames et al., 1999; Vaudry et al., 2010, 2015). These observations suggest that, in fish as in mammals, UII/URPs may act centrally and peripherally to control cardiovascular activity. Indeed, intracerebrovenricular (ICV) injection of UII and to a lesser extent URPs in the rainbow trout *Onchorynchus mykiss* (Le Mével et al., 1996; Vanegas et al., 2015) and in the eel *Anguilla japonica* (Nobata et al., 2011), and UII in mammals (Watson and May, 2004) elevates blood pressure but also accelerates heart rate. Since, in these studies, heart rate did not counter-regulate blood pressure elevation as might be expected, it appears that the cardiac baroreflex was impaired following ICV injection of UII/URPs. In mammals, the cardiovascular effects of peripherally administered UII are variable, depending upon the species used and the presence or absence of anesthesia, inasmuch as increase or decrease in blood pressure mostly associated with cardiac positive chronotropic action are observed in rats, sheep and monkeys (Watson and May, 2004). In trout and eel however, a consistent increase in the dorsal aortic blood pressure is observed following intra-arterial (IA) injection of UII and to a lesser extent URP1 but not URP2 (Le Mével et al., 1996; Nobata et al., 2011; Vanegas et al., 2015), but the baroreflexogenic bradycardia only occurs after UII injection in trout. Since the cardiac baroreflex response is an essential component of the cardiovascular regulation that buffers abrupt changes in blood pressure to maintain homeostasis (La Rovere et al., 2008; Olson, 2011), we decided to clarify the change in the cardiac baroreflex sensitivity (BRS) after ICV and IA injections of trout UII in our established trout model (Lancien et al., 2011). We contrasted the effects of UII with those of the two paralogs URP1 and URP2.

## Materials and methods

### Peptides and chemicals

Trout UII, zebrafish URP1, and URP2 (Waugh and Conlon, 1993; Tostivint et al., 2013) were synthesized as previously described (Chatenet et al., 2004; Lancien et al., 2004). Table 1 summarizes the physico-chemical characteristics of the synthetic peptides. The peptides were dissolved in Ringer's solution (vehicle) and stored in stock solutions at −25°C. Immediately before use, UII, URP1, or URP2 were diluted to the desired concentration with Ringer's solution. The composition of the Ringer's solution was (in mM): NaCl 124, KCl 3, CaCl_2_ 0.75, MgSO_4_ 1.30, KH_2_PO_4_ 1.24, NaHCO_3_ 12, glucose 10 (pH 7.8). All solutions were sterilized by filtration through 0.22 μm filters (Millipore, Molsheim, France) before injection.

**Table 1:** **Physico-chemical characteristics of the synthetized peptides**.

**Peptide**	**Sequence**	**RP-HPLC**		**MS**	
		**t_R_ (min)^a^**	**Purity (%)**	**Calcd.^b^**	**Obsd.^c^**
Trout UII	GGNSECFWKYCVTN	20.33	99.9	1389.55	1390.48
Zebrafish URP1	ACFWKYCVTN	19.40	96.6	1231.51	1232.69
Zebrafish URP2	VCFWKYCSQN	19.92	99.9	1274.52	1275.64

a *Retention time determined by RP-HPLC on a Vydac 218TP54 C18 column (0.46 x 25 cm) using a linear gradient (10–60% over 25 min) of acetonitrile/TFA (99.9:0.1, v/v) at a flow rate of 1 mL/min*.

b *Theorical monoisotopic molecular weight*.

c *m/z value assessed by MALDI-TOF-MS on a Voyager DE-PRO in the reflector mode with α-cyano-4-hydroxycinnamic acid as a matrix*.

### Animals

Adults rainbow trout *Oncorhynchus mykiss* (251 ± 26 g body wt, mean ± SEM, *n* = 98) of both sexes were purchased locally and transferred in a well-oxygenated and thermostatically controlled water tank to the laboratory. All fish were kept in a 1000-liter tank containing circulating dechlorinated and aerated tap water (11–12°C), under a standard photoperiod (lights on 09:00 AM–08:00 PM). The fish were allowed at least 3 weeks to acclimate under these conditions before the experiments were started. Animal manipulations and experimental protocols were approved by the Comité d'Ethique Finistérien en Expérimentation Animale (authorization number 02142.01).

### Experimental procedures

The surgical procedures have previously been described in detail (Lancien et al., 2004; Le Mével et al., 2012; Vanegas et al., 2015). Briefly, anesthetized rainbow trout were equipped with two electrocardiographic electrodes, a dorsal aortic cannula, an ICV microguide, and a buccal catheter that was used to record the buccal ventilatory pressure (not quantified in the present study). After surgery, the animals were transferred to a 6-liter blackened chamber supplied with dechlorinated and aerated tap water (10–11°C) that was both recirculating and through-flowing. Oxygen pressure within the water tank (Pw_O2_) and pH were continuously recorded and maintained at constant levels (Pw_O2_ = 20 kPa; pH = 7.4–7.6). The trout were allowed to recover from surgery and to become accustomed to their new environment for 48–72 h.

Each day, after dorsal aortic blood pressure (*P*_DA_) and heart rate were stabilized for at least 90 min, parameters were recorded for 30 min and the different injection protocols begun. The animals received no more than two ICV or IA injections of peptide per day with a delay of at least 5 h between the injections. Some trout received both ICV and IA injections, and in this case, the delay between the two injections was 1 day, and the type of injections was randomized among animals. No single fish was studied for more than 2 days and control experiments revealed that there was no significant change in performance over this period.

### Intracerebroventricular and intra-arterial administration of peptides

For the ICV protocols, the injector was introduced within the ICV guide prior to the beginning of a recording session which lasted 30 min. All injections were made at the 5 min of the test and the injector was left in place for a further 5 min to allow for complete diffusion of the agent and to minimize the spread of substances upwards in the cannula tract. The fish received first an ICV injection of vehicle (0.5 μl) and, 30 min later, an ICV injection of UII, URP1, or URP2 (5, 50 or 500 pmol in 0.5 μl). For IA injections, 5 min after the beginning of the recording session, 50 μl of vehicle, UII at doses of 5, 50 or 500 pmol, URP1 or URP2 at doses of 50 or 500 pmol was injected through the dorsal aorta and immediately flushed by 150 μl of vehicle.

### Data acquisition and analysis of cardiovascular variables

The ECG and *P*_DA_ signals were recorded using standardized electronic devices whose output were digitalized at 1000 Hz, visualized on the screen of a PC during the 30-min recording period and finally stored using PowerLab 4/30 data acquisition system (ADInstruments, Oxford, England) and LabChart Pro software (v.7.0; ADInstruments, Oxford, England) (Vanegas et al., 2015). ECG and *P*_DA_ signals were then processed off-line with custom-made programs written in LabView 6.1 (Laboratory Virtual Instrument Engineering Workbench, National Instruments, Austin, USA). Recordings were excluded from the analysis if they contained excessive artifacts on ECG and *P*_DA_ signals. For all protocols, the entire 25-min post-injection segments of ECG and *P*_DA_ signals were selected after ICV or IA injections of vehicle, UII, URP1, or URP2.

The R–R intervals (reflecting the time between each R wave of the ECG), systolic blood pressure (SBP) and cardiac baroreflex were calculated as previously described (Lancien et al., 2011). The R–R intervals were determined after detection of the R waves from the ECG recordings and SBP was identified from the pulsatile *P*_DA_. Their mean values were calculated. R–R interval and SBP time series were resampled at 2.56 Hz to obtain equidistant data points. The linear trend was removed from the new time series and 29 segments of 256 data points (100 s) overlapping by half were subjected to a Hanning window. To investigate to what extend the input signal, SBP, influences the output signal, the R–R interval, the coherence, phase and transfer function spectra of SBP against the R–R interval were determined (Lancien et al., 2011). A relatively high coherence between the two signals and a negative phase shift (SBP changes precedes R–R interval fluctuations) indicates that the SBP mediates the changes in the R–R intervals. The cardiac BRS was estimated as the mean of the gain of the transfer function when the coherence was high and the phase negative.

All calculations for R–R interval (in ms), SBP (in kPa), and cardiac BRS (in kPa/ms) were made for the entire post-injection period of 25 min and the results were averaged for trout subjected to the same protocol.

### Statistical analysis

Data are expressed as means + or ± SEM (standard error of the mean) on histograms or in percentage change in the text. For comparison between groups, the data were initially analyzed using a one-way ANOVA followed by the Dunnett's test for comparisons between vehicle-injected trout and trout receiving peptides. A value of *P* < 0.05 was considered significant. The statistical tests were performed and the graphs constructed using GraphPad Prism 5.0 (GraphPad, San Diego, CA).

## Results

### Cardiac baroreflex sensitivity to central UII, URP1, and URP2

**Figure 1:**
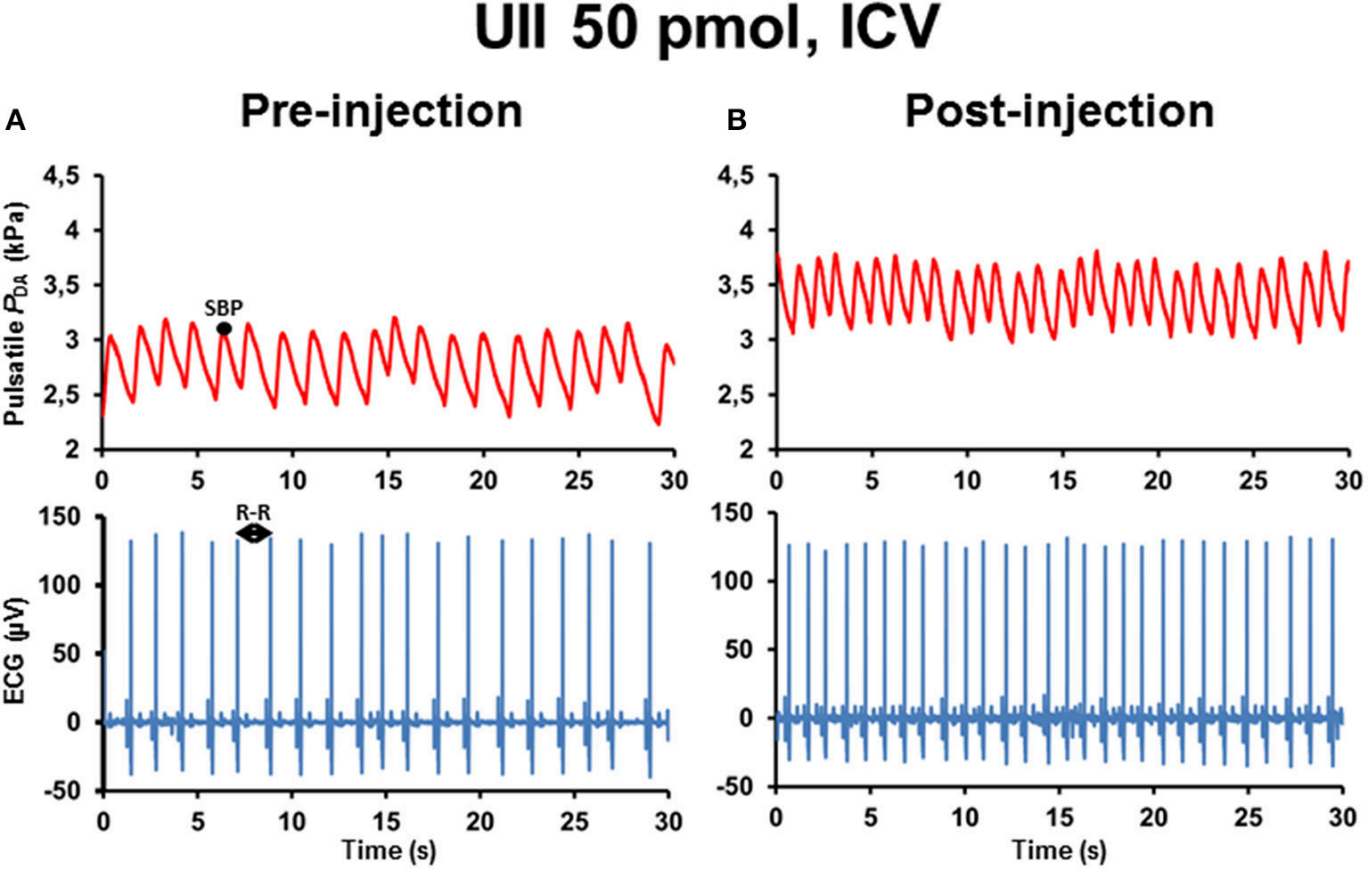
**Raw tracings of 30-s duration in a single unanesthetized trout illustrating the changes observed in pulsatile dorsal aortic blood pressure (***P***_**DA**_) and electrocardiographic (ECG) signals between the pre-injection period (A)** and the post-injection period **(B)** after intracerebroventricular (ICV) injection of 50 pmol UII. Note that, compared with the pre-injection period, ICV injection of UII produces an elevation of systolic blood pressure (SBP) but a decrease in the R–R interval of the ECG (reflecting a tachycardia).

Figure 1 illustrates 30-s recordings of pulsatile *P*_DA_ and ECG signals taken during the pre-injection period (Figure 1A) and during the post-injection period (Figure 1B) after ICV injection of 50 pmol UII. Comparison of the post-injection and the pre-injection signals revealed that UII caused a marked elevation in SBP associated with a sharp reduction of the R–R interval of the ECG.

**Figure 2:**
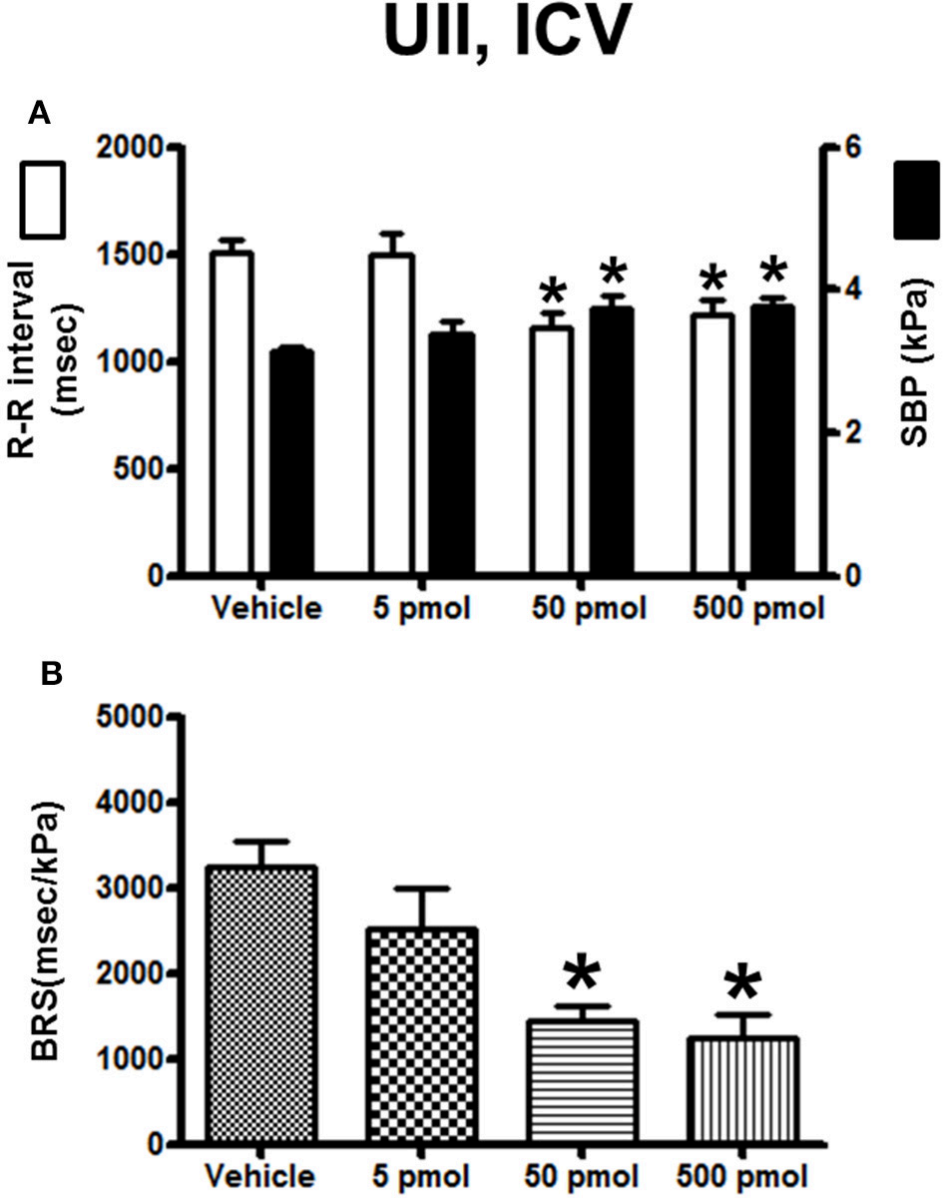
**Histograms showing (A)** the R–R intervals (scale on the left) and the SBP values (scale on the right), **(B)** the BRS during the 5–30 min period after intracerebroventricular injection of 0 (vehicle, *n* = 24), 5 pmol (*n* = 8), 50 pmol (*n* = 7) and 500 pmol (*n* = 9) UII. *n*, number of trout. ^*^P < 0.05 vs. vehicle.

The histograms in Figure 2 summarize the average changes in R–R interval and SBP (Figure 2A) and in BRS (Figure 2B) after ICV injection of vehicle or a range of doses (5–500 pmol) of UII. Compared with vehicle-injected trout, UII produced a gradual increase in SBP. The threshold dose for a statistically significant effect on SBP was 50 pmol and, at this dose, the R–R interval decreased significantly (Figure 2A). These effects on the two cardiovascular variables remained significant up to the 500-pmol dose (Figure 2A). Figure 2B demonstrates that the BRS was dose-dependently reduced following ICV injection of UII. The threshold dose for a statistically significant effect on BRS was 50 pmol (−55%; 1450 ± 165 ms/kPa vs. 3240 ± 300 ms/kPa; *P* < 0.05) and the maximum decrease was observed for the 500-pmol dose.

**Figure 3:**
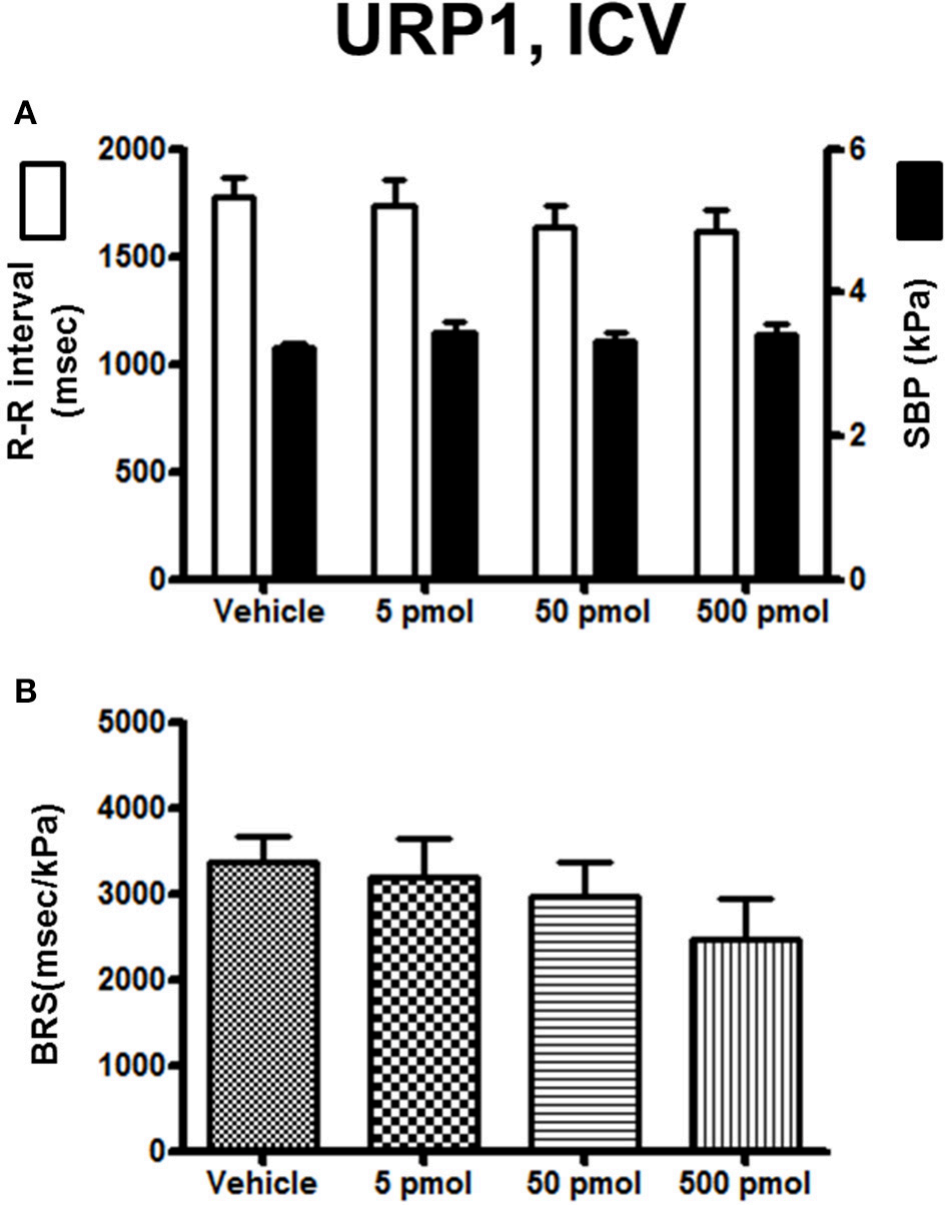
**Histograms showing (A)** the R–R intervals (scale on the left) and the SBP values (scale on the right), **(B)** the BRS during the 5–30 min period after intracerebroventricular injection of 0 (vehicle, *n* = 20), 5 pmol (*n* = 9), 50 pmol (*n* = 11) and 500 pmol (*n* = 10) URP1. *n*, number of trout.

**Figure 4:**
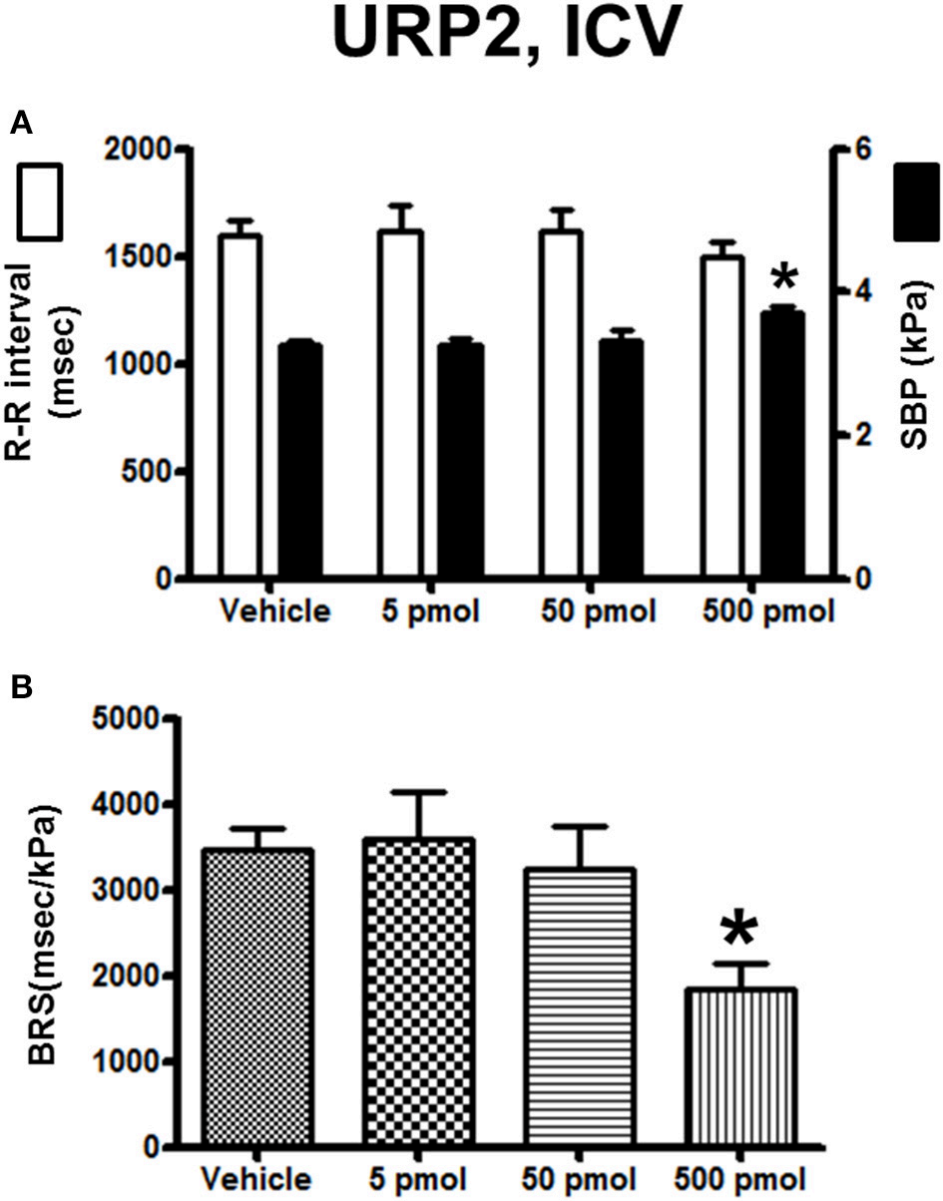
**Histograms showing (A)** the R–R intervals (scale on the left) and the SBP values (scale on the right), **(B)** the BRS during the 5–30 min period after intracerebroventricular injection of 0 (vehicle, *n* = 30), 5 pmol (*n* = 13), 50 pmol (*n* = 14) and 500 pmol (*n* = 9) URP2. *n*, number of trout. ^*^P < 0.05 vs. vehicle.

The effect of URP1 and URP2 on the cardiovascular variables and cardiac BRS are summarized in Figures 3, 4, respectively. In contrast to UII, ICV injection of URP1 provoked no significant change in SBP and the R–R interval (Figure 3A). URP1 tended to reduce BRS but this effect was not statistically significant even at the highest dose (Figure 3B). Only the 500-pmol dose of URP2 caused a significant increase in SBP without changing significantly the R-R interval (Figure 4A) but, at this 500-pmol dose, URP2 evoked a significant depression in BRS (−47%, 1850 ± 290 ms/kPa vs. 3470 ± 240 ms/kPa, *P* < 0.05; Figure 4B).

### Cardiac baroreflex sensitivity to peripheral UII, URP1, and URP2

**Figure 5:**
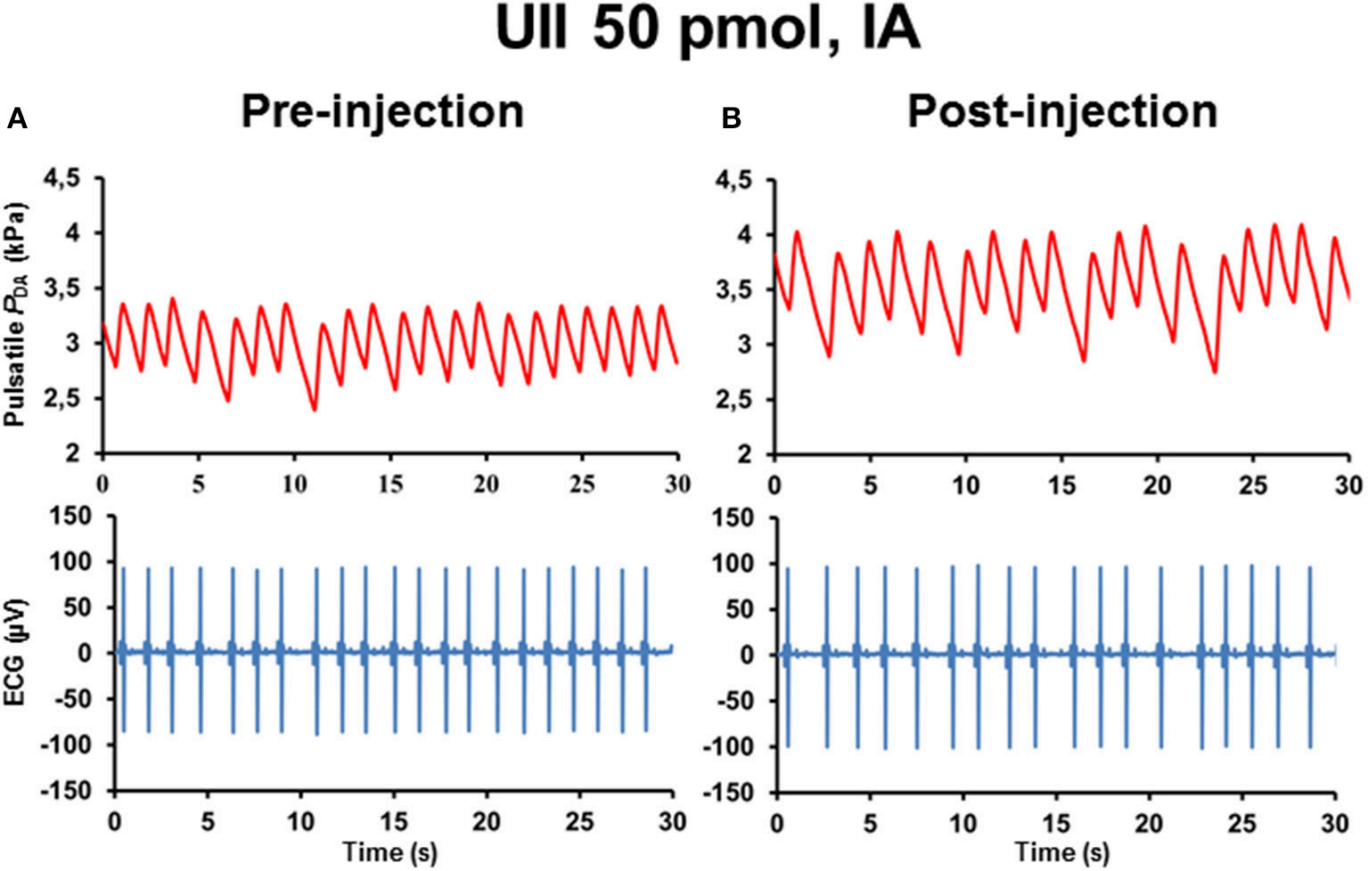
**Raw tracings of 30-s duration in a single unanesthetized trout showing the changes observed in pulsatile dorsal aortic blood pressure (***P***_**DA**_) and electrocardiographic (ECG) signals between the pre-injection period (A)** and the post-injection period **(B)** after intra-arterial (IA) injection of 50 pmol UII. Note that, compared with the pre-injection period, IA injection of UII provokes an increase in SBP and in the R–R interval of the ECG (reflecting a bradycardia).

Figure 5 illustrates 30-s recordings of pulsatile *P*_DA_ and ECG signals taken during the pre-injection period (Figure 5A) and during the post-injection period (Figure 5B) after IA injection of 50 pmol UII. Comparison of the post-injection and the pre-injection signals revealed that UII caused a marked elevation in SBP associated with a potent increase in the R–R interval of the ECG.

**Figure 6:**
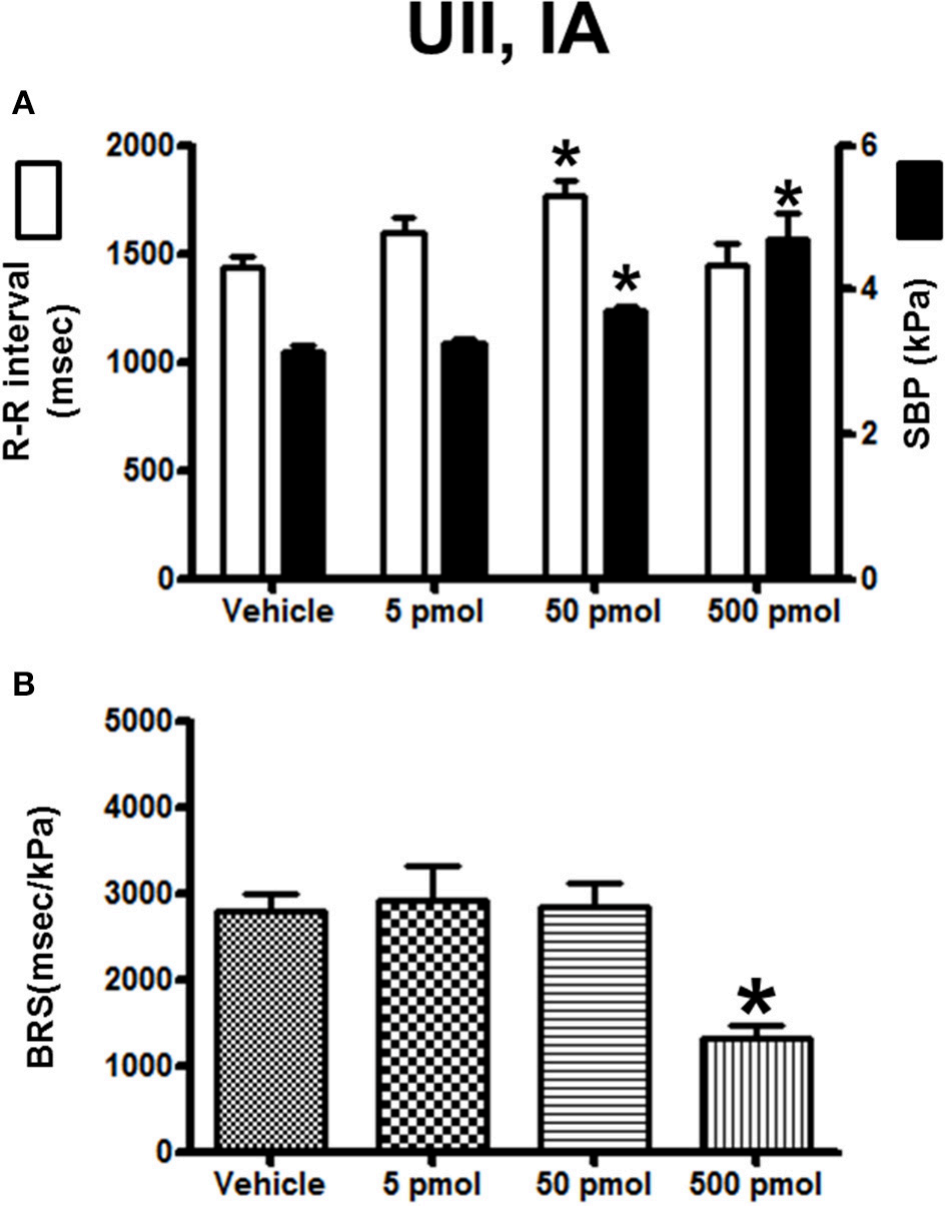
**Histograms showing (A)** the R–R intervals (scale on the left) and the SBP values (scale on the right), **(B)** the BRS during the 5–30 min period after intra-arterial injection of 0 (vehicle, *n* = 20), 5 pmol (*n* = 6), 50 pmol (*n* = 10) and 500 pmol (*n* = 7) UII. *n*, number of trout. ^*^P < 0.05 vs. vehicle.

The effects of IA injection of vehicle or a range of doses of UII (5–500 pmol) on the cardiovascular variables and BRS are summarized in Figure 6. UII provoked a clear dose-dependent increase in SBP with a threshold dose of 50 pmol for a significant effect and a maximum hypertension at the 500-pmol dose (Figure 6A). In marked contrast with the response observed after ICV injection, the R–R interval increased up to the 50-pmol dose and then returned to baseline level at the 500-pmol dose (Figure 6A). Interestingly, and compared to vehicle-injected trout, there was no change in the BRS using the lowest picomolar doses of UII (50 pmol; 2530 ± 270 ms/kPa vs. 2600 ± 180 ms/kPa; *P* < 0.05) but the highest dose of UII caused a 2-fold decrease in the BRS (Figure 6B).

**Figure 7:**
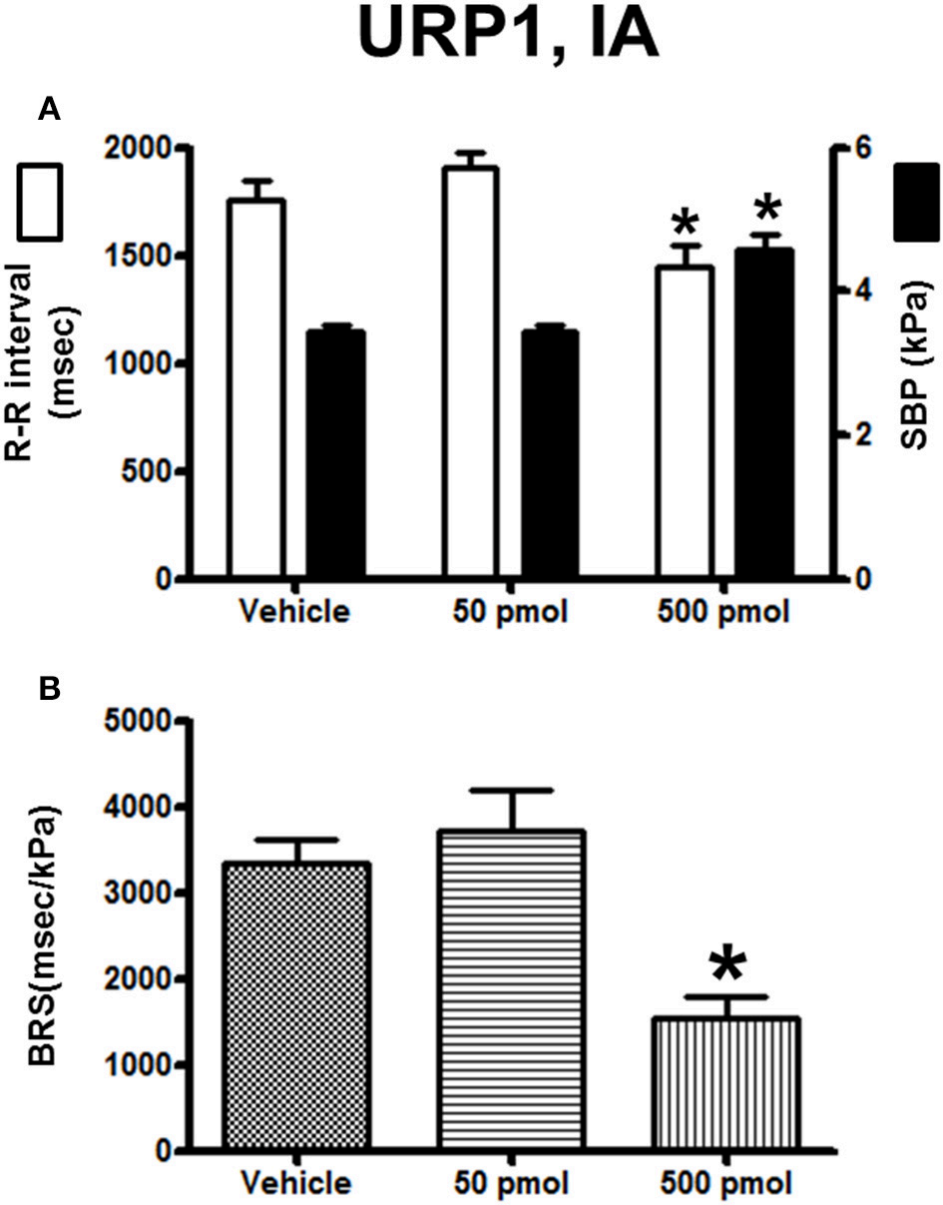
**Histograms showing (A)** the R–R intervals (scale on the left) and the SBP values (scale on the right), **(B)** the BRS during the 5–30 min period after intra-arterial injection of 0 (vehicle, *n* = 19), 50 pmol (*n* = 14) and 500 pmol (*n* = 11) URP1. *n*, number of trout. ^*^P < 0.05 vs. vehicle.

**Figure 8:**
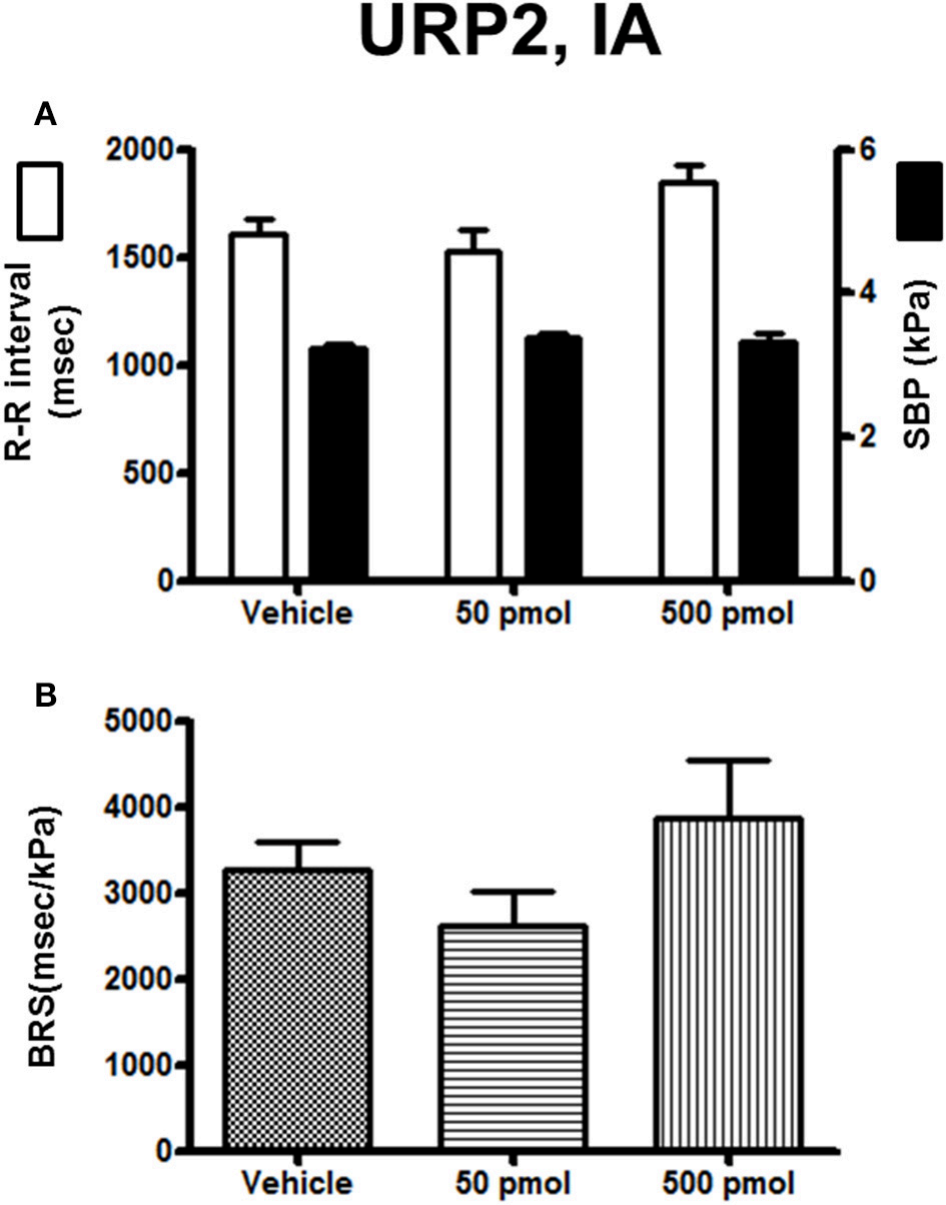
**Histograms showing (A)** the R–R intervals (scale on the left) and the SBP values (scale on the right), **(B)** the BRS during the 5–30 min period after intra-arterial injection of 0 (vehicle, *n* = 19), 50 pmol (*n* = 11), and 500 pmol (*n* = 9) URP2. *n*, number of trout. ^*^P < 0.05 vs. vehicle.

The effect of URP1 and URP2 on the cardiovascular variables and cardiac BRS are depicted in Figures 7, 8, respectively. Only the highest dose of URP1 (500 pmol) elevated SBP but decreased the R–R interval (Figure 7A). The attenuation of the BRS was only significant after IA injection of this highest dose of URP1 (−53%; 1540 ± 250 ms/kPa vs. 3330 ± 290 ms/kPa, *P* < 0.05; Figure 7B). The IA injection of URP2 was devoid of effect on the cardiovascular variables and BRS (Figure 8).

## Discussion

Our study represents the first attempt in any animal species to quantify the changes in spontaneous cardiac BRS after ICV and IA injections of UII and URPs. The inhibitory effect of UII on the BRS after ICV injection of low picomolar doses, and its absence of effect after peripheral injection of equimolar doses suggests that only the central urotensinergic system is involved in the attenuation of the BRS. In addition, the limited and quite divergent BRS effects of the two structurally UII-related peptides URP1 and URP2, that share the cyclic hexapeptide core sequence of UII but differ in the N- and C-terminal regions (Table 1), emphasize the importance of the amino-acid residues flanking the N- and C-terminus of the cyclic region of the fish UII-molecule for full interaction with the fish UT receptor. This observation also indicates that the action of UII is specific for this peptide.

The baroreflex has been conserved across vertebrate's evolution (Bagshaw, 1985; Van Vliet and West, 1994) and, in fish as in mammals, the spontaneous BRS can be measured by means of cross spectrum analysis of R–R interval and SBP variabilities (Head et al., 2001; La Rovere et al., 2008; Lancien et al., 2011). This technique offers the great advantage to prevent the use of any stressful surgical interventions for loading or unloading the baroreceptors and circumvents the use of vasoactive drugs to evoke a baroreflex response, drugs that may interfere with baroreflex functioning. However, using this approach, we assessed only one aspect of the baroreflex loop, i.e., the baroreflex regulation of heart rate, but not the baroreflex regulation of vascular tone.

### Central effects of UII and URPs

In fish, the primary baroreceptor sites are the gills (Ristori and Dessaux, 1970; Nilsson and Sundin, 1998; Armelin et al., 2016). Afferent baroreceptive activity runs along the glossopharyngeal (IXth cranial nerve) and along the vagus (Xth cranial nerve) to reach the medulla oblongata. Little is known regarding the central neuroregulatory mechanisms involved in the baroreflex responses in fish, except that glutamatergic pathways within the caudal part of the nucleus tractus solitarii (NTS) play a key role to transmit baroreceptive information to the dorsal vagal motor nucleus (DVMN) and hence, to control the cardiac vagal outflow (Taylor et al., 1999; Sundin et al., 2003). In trout, we previously demonstrated that spontaneous increases or decreases in SBP provoke a bradycardia or a tachycardia, respectively, that are exclusively mediated by the parasympathetic nervous system and thus that the spontaneous BRS can be considered as an index of parasympathetic activity to the heart (Lancien and Le Mével, 2007). The level at which UII/URPs upon ICV injection mimic the possible action of the endogenous urotensinergic system(s) on the neural networks involved in the baroreflex response in trout, and notably on its cardio-vagal inhibitory component, cannot be established from the present experiments. However, some working hypotheses can be proposed. Since the exogenous peptides were injected into the third ventricle at the level of the nucleus preopticus, a nucleus homologous to the paraventricular nucleus of mammals, it is reasonable to speculate that UII/URPs might primarily affect the activity of preoptic neuropeptidergic neurons like arginine vasotocin and isotocin neurons that project toward critical cardiovascular brainstem nuclei including the NTS and the DVMN (Batten et al., 1990; Saito et al., 2004). In addition, the urotensin peptides may diffuse within the cerebrospinal fluid toward the fourth ventricle to control the activity of these cardiovascular nuclei. Since we previously demonstrated that, after peripheral injection of UII, bradycardia may arise from adrenergic-mediated activation of the cardio-inhibitory baroreflex (Le Mével et al., 1996), we can speculate that the reduced BRS after ICV injection of UII may also be due to blockage of central adrenergic pathways. Neuroanatomical and molecular data from various teleost species provide some clues for these hypotheses. UT is expressed in the teleost brain (Lu et al., 2006) and UII immunoreactivity and URP2 expression are seen in the region surrounding the fourth ventricle (Yulis and Lederis, 1988; Parmentier et al., 2011; Quan et al., 2015) while URP1 is notably expressed in the NTS and the glosso-pharyngeal motor nuclei (Nobata et al., 2011; Quan et al., 2015). Whether UII/URP neurons present within the brainstem contribute more precisely to the control of the BRS by interacting with baroreflex afferent inputs at the level of the NTS remains to be elucidated.

Our results demonstrating that third ventricle injection of native UII in trout causes an attenuation of the BRS can be compared with previous cardiovascular studies conducted with UII in mammals. As previously mentioned, in normotensive and hypertensive unanesthetized rats (Lin et al., 2003) and in unanesthetized sheep (Watson et al., 2003), ICV administration of UII causes pressor and tachycardic responses through activation of the sympathetic system indicating that, in these species, also the cardiac baroreflex response is impaired. Studies conducted on unanesthetized sheep to test this hypothesis demonstrated that, after ICV infusion of UII, the cardiac baroreflex response is effectively blunted since no changes occur in the cardiac sympathetic nerve activity in spite of an increase in blood pressure (Hood et al., 2005). Since, in sheep, ICV UII also stimulates the sympatho-adrenal axis resulting in elevation of plasma epinephrine (Watson et al., 2003) and since propranolol blocks UII-induced tachycardia (Hood et al., 2005), an increase in plasma level of epinephrine together with preservation of cardiac sympathetic nerve activity levels was postulated to be responsible for the chronotropic effect of centrally administered UII (Hood et al., 2005). Consequently, the cardiac baroreflex response to an increase in blood pressure after ICV injection of UII, in mammals, is probably impaired through the inability of central baroreflex networks to drive sufficient vagal cardiac inhibitory influx and to block cardiac sympathetic activity. It is known that, in mammals, the central cardiomodulatory action of UII is site-dependent as local administration of UII in discrete brain nuclei produces differential heart rate responses (Lin et al., 2003). Interestingly, a recent study in conscious rat demonstrates that micro-injection of UII within the nucleus ambiguus, a key site controlling parasympathetic cardiac tone, elicits a bradycardia, indicating the involvement of UII in controlling vagal outflow (Brailoiu et al., 2014). The regulation of the baroreflex in fish is probably as complex as in mammals, and further studies are required to determine more precisely the impact of UII/URPs on the cardio-vagal component of the baroreflex, as well as the potential effects of these peptides on sympathetic outflow to cardiovascular and chromaffin tissues. We previously demonstrated that, after ICV injection of native neuropeptides in trout, angiotensin II (Lancien and Le Mével, 2007), pituitary adenylate cyclase-activating polypeptide and vasoactive intestinal peptide (Lancien et al., 2011) decrease BRS. The present study adds UII and to a lesser extend URP2 as new candidates acting centrally as neuromodulators or neurotransmitters to control the cardiac baroreflex.

### Peripheral effects of UII and URPs

The bradycardic response to UII-induced hypertension is parasympathetically-mediated since, in atropinized trout, this bradycardia is abolished (Vanegas et al., 2016). In the present study, ICV or IA administration of UII at its lowest doses caused similar hypertension but in contrast to its ICV effects, peripheral UII provoked a bradycardia and the BRS was not disturbed. These data support the idea that, after peripheral administration, UII does not affect vagal feedback gain on the heart. Nonetheless, a significant decrease in BRS was observed with the largest dose of UII. Since the BRS reduction to the highest dose of IA administered UII was very similar to that observed after ICV injection of the same dose, we assume that this effect was mediated through a neurogenic pathway after diffusion of UII to critical target sites in the brain. However, because UT is also strongly expressed in the teleost heart (Lu et al., 2006), we cannot exclude a possible direct positive chronotropic effect of large doses of UII counter-acting the baroreflex response. According to the route of administration the highest dose of URP1 and URP2 had opposite effect on the BRS, URP2 being more efficient than URP1 in the brain and inversely at the periphery. It remains to be determined whether these opposite effects of URP1 and URP2 can be ascribed to differential interaction with UT or to binding to distinct UT subtypes. Moreover, since the two URPs are exclusively expressed in the CNS, the physiological significance of URP1 on BRS remains also to be ascertained.

### Possible physiological significance

In humans, a decrease in the BRS is associated with hypertension and cardiovascular tissue damages (La Rovere et al., 2008). In fish, the essential role of the baroreflex is to prevent damage to organs primarily at risk, such as the delicate respiratory vasculature of the gills (Bagshaw, 1985). Consequently, endogenous UII, a peptide that provokes inhibition of BRS after central exogenous injection, may contribute to exacerbate the increase in blood pressure and may have deleterious effects. In the periphery, UII also causes hypertension but the maintenance of the BRS may be beneficial to prevent excessive elevation of blood pressure. This hypertension might be useful to correct hypotensive situations in order to maintain tissue perfusion pressure. Further studies are required to elucidate the site(s) and mechanisms of action of UII/URPs on the baroreflex pathways in trout and to determine under which circumstances the central and peripheral urotensinergic systems are recruited to regulate blood pressure.

In conclusion, our study has shown that UII and to a lesser extent URPs interact with CNS blood pressure-regulating structures, not only to elevate blood pressure and heart rate but also to reduce BRS. Conversely, at the periphery, UII at low picomolar doses increases blood pressure and decreases heart rate but does not alter BRS.

## Author contributions

FL, GV, and JM, performed the experiments. JL synthetized the peptides. FL analyzed the data. FL and JM, wrote the manuscript. GV, JL, and HV, edited and revised critically the manuscript. All authors approved the final version of the manuscript.

### Conflict of interest statement

The authors declare that the research was conducted in the absence of any commercial or financial relationships that could be construed as a potential conflict of interest.

## Abbreviations

BRS: baroreflex sensitivity
CNS: central nervous system
DVMN: dorsal vagal motor nucleus
ECG: electrocardiographic
IA: intra-arterial
ICV: intracerebroventricular
NTS: nucleus tractus solitarii
*P*_DA_: dorsal aortic blood pressure
SBP: systolic blood pressure
UII: urotensin II
URP: urotensin II-related peptide
URP1: urotensin II-related peptide 1
URP2: urotensin II-related peptide 2
UT: urotensin II receptor.
